# Whole-genome sequencing-based pathogen characterization for streptococcal infection directly from positive blood culture samples

**DOI:** 10.1128/jcm.01126-25

**Published:** 2025-12-08

**Authors:** Yuan Li, Wuling Lin, Zhongya Li, Theresa Tran, Benjamin J. Metcalf, Srinivasan Velusamy, Annastasia Gross, Paula Snippes Vagnone, Ruth Lynfield, Bernard Beall, Lesley McGee, Sopio Chochua

**Affiliations:** 1 Division of Bacterial Diseases, National Center for Immunization and Respiratory Diseases, Centers for Disease Control and Prevention, Atlanta, Georgia, USA; 2 ASRT Inc., Atlanta, Georgia, USA; 3 Minnesota Department of Health 11055, St Paul, Minnesota, USA; Children's Hospital Los Angeles, Los Angeles, California, USA

**Keywords:** public health surveillance, whole-genome sequencing, blood culture, streptococcal disease

## Abstract

Clinical laboratories are increasingly using diagnostic tests directly on positive blood cultures, which may lead to fewer attempts to recover bacterial isolates. Consequently, public health laboratories can benefit from assays that directly process blood culture samples without requiring submission of clinical isolates to determine additional pathogen features not identified by clinical tests, such as vaccine serotype and bacterial genomic relatedness, for surveillance and outbreak response purposes. In partnership with the Minnesota Active Bacterial Core surveillance (ABCs) site, we identified blood culture samples positive for ABCs streptococcal pathogens and characterized them by a direct whole-genome sequencing from blood culture (dWGS) assay. The dWGS results were compared with the results of a reference method (WGS of isolates from the same cultures) to evaluate concordance in pathogen features and genome assemblies. Of the 97 eligible blood culture samples, 83 (86%) passed dWGS quality control criteria and were subjected to a total of 655 dWGS-based tests, which yielded 651 (99.3%) evaluable results. The percent agreement with reference results was 100% (83/83) for M protein gene (*emm*)/capsular types and 100% (81/81) for multilocus sequencing types. For genotypic antimicrobial susceptibility testing prediction, the percent prediction agreement was 100% (487/487), false resistant prediction rate was 0% (0/417), and the false susceptible prediction rate was 0% (0/66). Assemblies of pathogen genomes from the same patient differed by 1.08 ± 1.68 (mean ± SD) sites per genome. The dWGS assay can extract high-quality, important streptococcal strain characteristics directly from positive blood culture samples to support evolving public health needs.

**IMPORTANCE:**

Whole-genome sequencing (WGS) technologies have emerged as a transformative toolkit used by public health microbiology laboratories to detect and characterize pathogens. The surveillance of bacterial diseases often relies on clinical laboratories to submit pathogen isolates to regional or national public health laboratories, which have the capacity to routinely conduct WGS-based strain characterization. Clinical laboratories are increasingly using diagnostic tests directly on positive blood cultures, which may lead to fewer attempts to recover bacterial isolates. The study evaluated a direct whole-genome sequencing from blood culture (dWGS) assay that directly processes blood culture samples. The dWGS assay recovered high quality, important streptococcal strain characteristics, including vaccine serotypes and whole-genome assemblies, without requiring submission of clinical isolates. Thus, the dWGS assay represents a promising tool for addressing the evolving needs of public health laboratories in the metagenomics era.

## INTRODUCTION

Whole-genome sequencing (WGS) technologies have emerged as a transformative toolkit used by public health microbiology laboratories to detect and characterize pathogens (1, 2). Commonly used next-generation sequencing (NGS) platforms sequence millions to billions of DNA fragments in parallel and acquire high-resolution information of the pathogen genome at the single nucleotide level (3). There has been expansion in the use of NGS platforms in the past decade, making them widely available in public health laboratories. Bioinformatics analyses of WGS data can generate a comprehensive view of pathogen characteristics, including species identity, strain type, antimicrobial resistance (AMR), and virulence-related genes (4–6). Pathogen characterization is critical to support disease surveillance, track AMR trends, identify emerging lineages, and inform prevention strategies (7, 8). WGS data are also used to accurately determine pathogen genomic relatedness in suspected disease clusters, which improves outbreak detection and response (9).

Public health surveillance of bacterial diseases often relies on clinical laboratories to submit pathogen isolates to regional or national public health laboratories, which have the capacity to routinely conduct WGS-based strain characterization (10, 11). Recently, clinical laboratories are increasingly using diagnostic platforms that do not require pure bacterial isolate from positive blood culture samples, such as mass spectrometer systems (e.g., VITEK MS, MBT Sepsityper) and multiplex nucleic acid-based test systems (e.g., Biofire, Verigene), to expeditiously identify microorganisms (12, 13). These clinical tests usually do not provide detailed pathogen features, and this approach could reduce the availability of isolates for characterization by public health laboratories (14, 15). It is therefore imperative for public health laboratories to explore and adopt new assays that can analyze blood culture samples to provide vital strain information needed for disease surveillance, vaccine target assessment, outbreak response, and AMR tracking.

In this study, we developed a direct whole-genome sequencing from blood culture (dWGS) assay to characterize bacterial pathogens in blood culture samples. The dWGS results derived from each patient’s blood culture bottle sample were compared with the standard isolate-based WGS strain characterization results derived from the corresponding patient’s blood culture isolate to evaluate assay performance as well as genome assembly accuracy.

## MATERIALS AND METHODS

### Bacterial isolates and blood culture samples

Group A *Streptococcus* (GAS), Group B *Streptococcus* (GBS), and *Streptococcus pneumoniae* (SPN) isolates were identified from the Active Bacterial Core surveillance (ABCs) by the Minnesota Department of Health. ABCs is a laboratory- and population-based system that actively monitors five invasive bacterial pathogens, including GAS, GBS, *Haemophilus influenzae*, *Neisseria meningitidis*, and SPN, across 10 surveillance sites in 10 US states (7). A case is defined as a resident of the surveillance area with the detection of an ABCs pathogen in a specimen from a normally sterile site (11). For all ABCs cases identified, CDC receives bacterial isolates from ABCs site microbiology laboratories for additional characterization as part of routine surveillance. For this study, the Minnesota site identified blood culture samples that were positive for GAS, GBS, or SPN by an isolation-independent test (Biofire or Verigene) or a routine identification test (e.g., MALDI-TOF) among ABCs cases and provided aliquots from blood culture bottles (≥2.5 mL) to CDC for isolation-independent pathogen characterization. The positive cultures were also subcultured to generate isolates as part of routine surveillance. Blood culture systems used included the BACTEC blood culture system (Becton, Dickinson and Company, Franklin Lakes, New Jersey, USA) and the BACT/ALERT VIRTUO blood culture system (bioMérieux, Marcy l'Étoile, France). Blood culture samples were stored at <–70°C until processing. The number of paired blood culture samples and isolates tested were 15, 44, and 38 for GAS, GBS, and SPN, respectively.

Activities of the ABCs network are considered part of public health surveillance, and informed consent is not required by CDC’s Institutional Review Board.

### Reference assay for pathogen characterization

GAS, GBS, and SPN isolates were subjected to WGS-based strain characterization using standard ABCs procedures as described before (4–6). Briefly, colony-purified isolates were cultured in Todd Hewitt Broth supplemented with Yeast Extract (THY) medium (CDC Division of Scientific Resources, Atlanta, GA, USA) for DNA isolation. DNA was extracted with the QIAamp DNA Mini Kit (QIAGEN, Germantown, Maryland, USA), and libraries were prepared for sequencing on an Illumina MiSeq (Illumina, San Diego, CA, USA) platform using the sparQ DNA library kit (Quantabio, Beverly, MA, USA) using a PCR-free protocol. Pooled libraries were sequenced on the MiSeq instrument with 250 bp paired-end reads. Quality of reads was examined with FastQC version 0.11.5, and cutadapt version 1.8.3 was used for trimming adapter sequences and low-quality bases (parameters: -q 20; --minimum-length 50). For each isolate, a draft whole genome assembly was generated from WGS reads using VelvetOptimiser version 2.2.6. In this study, all trimmed reads with minimum base Phred scores ≥20 were used for genome assembly, with an average sequencing depth typically greater than 30-fold. The quality metrics of draft genome assembly, including number of contigs, contig N50 (the length of the smallest contig in the set that contains fewest and longest contigs whose combined length is at least 50% of the assembly), and assembly length (total number of bases in the assembly), was determined using the QUAST package (16). The criteria for passing WGS quality control (QC) in this study were a maximum contig number of 500, a minimum N50 of 10,000 bases, and an assembly length between 1.5 M and 2.5 M in the draft assembly. For samples that passed WGS QC, we further analyzed the WGS reads using previously validated, pathogen-specific bioinformatics pipelines (4–6) to obtain strain characterization results, including *emm* types (GAS)/capsular serotypes (GBS, SPN), multi-locus sequencing typing (MLST, all three pathogens), and genotypic antimicrobial susceptibility testing (AST) prediction of 5–7 clinically relevant antibiotics for each pathogen (Table S1). The genotypic AST prediction results are publicly available in surveillance reports (7).

Pipelines using VelvetOptimiser for assembly have been approved for ABCs strain characterization through institutional review of validation protocols and results, which were benchmarked against phenotypic testing. Bridging studies to transition to newer assemblers, such as SPAdes (17), are currently in progress. Genotypic AST predictions for ABC isolates were directly compared with phenotypic AST results to demonstrate accuracy in previous publications. These included evaluation of 1,454 GAS isolates (18) and an additional 591 isolates (19), 1,975 GBS isolates (20), and 2,316 SPN isolates (4). Furthermore, 2,528 SPN isolates (21) and an additional 1,781 isolates (22) were used specifically to validate prediction of minimum inhibitory concentrations for six β-lactam antibiotics.

### dWGS assay for pathogen characterization

Blood culture samples positive for GAS, GBS, or SPN were subjected to the dWGS assay for strain characterization. Total DNA was extracted from the blood culture samples using the QIAamp DNA Mini Kit (QIAGEN, Germantown, Maryland, USA) according to manufacturer’s instruction. Extracted DNA was evaluated by real-time PCR assays to confirm species identification by detecting pathogen-specific target genes (GAS: *spy*; GBS: *cfb*; SPN: *lytA*) according to previously reported methods (23–25). The DNA samples were then subjected to WGS using the same protocols as those used in the reference assay. After generating the WGS reads file, we removed all reads identified as potentially of human origin by using the STAT Human Sequence Removal Tool (26). The remaining non-human reads were processed by bioinformatics procedures identical to those used in the reference assay to obtain draft genome assembly, WGS QC metrics, and strain characterization results.

### Comparison between reference WGS (isolate-based) and dWGS results

The main objective was to evaluate agreement between dWGS and reference WGS assay (isolate-based) for strain characteristics that had evaluable results from both assays. For strain typing assays (*emm* typing, serotyping, and MLST), an evaluable result is one of the known types documented in the reference databases of the bioinformatics pipelines. For genotypic AST prediction, an evaluable result is one of the following: susceptible (S), intermediate (I), or resistant (R). Obtaining an evaluable result indicates test completion. A non-evaluable result is any test result other than an evaluable result, such as a potentially new MLST (“NEW”), *emm* type/capsular serotype gene target sequence not found (“NF”), or a new antimicrobial resistance gene allele that requires confirmative phenotypic testing (“FLAG”). A non-evaluable result indicates that further analyses are needed on a case-by-case basis.

For strain typing assays (*emm* type and capsular serotypes, MLST), we calculated the overall percent agreement between the dWGS and the reference results. Further, for each specific type *i* identified in a reference typing assay, we calculated the type-specific positive percent agreement (PPA*_i_*) and negative percent agreement (NPA*_i_*) using the following two steps. First, each dWGS typing result was assigned to only one of the following four categories:

TP*_i_* (true positive for type *i*): the dWGS result is i, and the reference result is i;

TN*_i_* (true negative for type *i*): the dWGS result is not i and the reference result is not i;

FP*_i_* (false positive for type *i*): the dWGS result is i and the reference result is not i;

FN*_i_* (false negative for type *i*): the dWGS result is not i and the reference result is i.

Second, the PPA*_i_* was calculated as (TP*_i_* count)/ (TP*_i_* count + FN*_i_* count) × 100% and NPA*_i_* as (TN*_i_* count)/(TN*_i_* count + FP*_i_* count) × 100%. Additionally, the overall PPA or sensitivity was then estimated as PPA=($\sum_{iЄI}TPicount$) / ($\sum_{iЄI}TPicount$ + $\sum_{iЄI}FNicount$) × 100% and overall NPA or specificity as NPA=($\sum_{iЄI}TNicount$)/ ($\sum_{iЄI}TNicount$ + $\sum_{iЄI}FPicount$) × 100%, where I represents all types identified in reference assay.

For genotypic AST prediction, we calculated percent prediction agreement (PA; agreement of categorical results (S, I, or R) between the dWGS and the reference assay), false intermediate prediction (FIP; a categorical result discrepancy in which either the reference or the dWGS result is I) rate, false resistant prediction (FRP; the reference result is S and the dWGS result is R) rate, and false susceptible prediction (FSP; the reference result is R, and the dWGS result is S) rate according to similar definitions in a previously published guidance (27).

For genome assemblies that passed WGS quality control, we determined the number of single nucleotide polymorphisms (SNPs) between the dWGS assembly from patient blood culture sample and the reference assembly from isolate of the same patient using the kSNP software (28).

For each sample, *emm* typing/serotyping, MLST, and genotypic AST prediction are separate bioinformatic analyses based on the same sequencing data. They are treated as separate tests to allow the statistical comparisons described above.

### Statistical analysis

The exact binomial two-sided 95% confidence intervals (CIs) were calculated for proportions according to the Clopper-Pearson method (29) implemented in the R stats::binom.test function (30). For strain typing assays (*emm* typing, serotyping, and MLST), the performance goals were at least 90% PA with at least an 80% lower bound of the 95% CI. For genotypic AST prediction, the performance goals were at least 90% PA, at most 3% FRP rate, and an upper 95% confidence limit for the true FSP rate of <7.5% and the lower 95% confidence limit for the true FSP rate of <1.5% (27). Genome assembly metrics (number of contigs, N50, and assembly length) were compared between dWGS and reference by the Wilcoxon signed-rank test, and multiple comparisons were adjusted using the Benjamini and Hochberg method implemented in the R stats:: p.adjust function (30). All analyses were conducted using R version 4.4.0 (30). WGS data have been deposited at the National Center for Biotechnology Information Sequence Read Archive with accession numbers PRJNA395240 (GAS), PRJNA355303 (GBS), and PRJNA284954 (SPN).

## RESULTS

**Fig 1:**
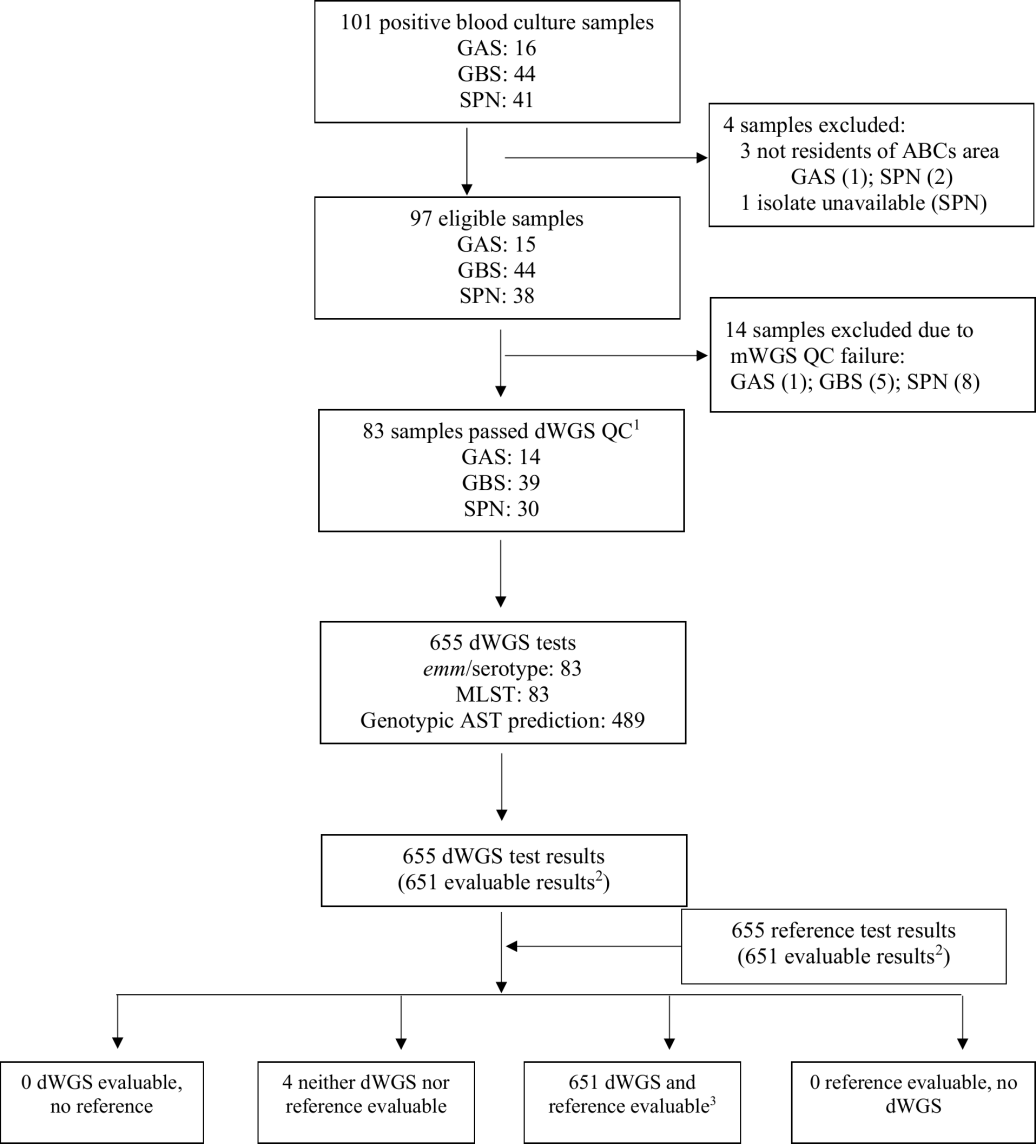
STARD diagram of the blood culture samples and testing. The dWGS analysis of the blood culture sample was compared with the isolate-based WGS analysis as the reference test. STARD = Standards for Reporting of Diagnostic Accuracy. ^1^The quality control (QC) success criteria are (i) a draft genome assembly with a contig number ≤500; (ii) assembly N50 ≥ 10,000; and (iii) total number of bases in the assembly between 1.5 M and 2.5 M. ^2^An evaluable result is a strain feature that is designed to be detected by the test. For *emm* typing/capsular serotyping and MLST, an evaluable result is one of the known types documented in the respective reference databases. For AST, an evaluable result is one of the following: susceptible (S), intermediate (I), or resistant (R). Obtaining an evaluable result indicates test completion. A non-evaluable result is any test result other than an evaluable result. Obtaining a non-evaluable result indicates test incompletion and warrants further investigation. ^3^These results were included in the main analysis.

From April 29, 2021, to August 15, 2022, 97 blood culture samples were identified by the Minnesota ABCs site to be eligible for this study. DNA extraction from blood culture samples was performed at 120 ± 55 (mean ± SD) days after the positive culture date. All 97 DNA samples were confirmed by real-time PCR to be positive for the targeted pathogens (Table S2) and were processed for WGS. Table S3 shows the number of human reads removed from each sample after sequencing. The median proportion of reads removed was 37.9% (interquartile range: 19.2%–63.9%). After QC, 83 (86%) samples proceeded to the dWGS-based testing (Fig. 1). The other 14 samples were excluded due to failing to pass the dWGS QC criteria. The 83 blood culture samples were subjected to 655 dWGS-based tests (Table S1), and for 651 tests (99.3%) evaluable results were obtained from both dWGS-based test and the reference method. The patient characteristics of the 83 ABCs cases whose blood culture samples were subjected to dWGS-based testing are shown in Table S4. Overall, 31 (37.3%) of the 83 cases were female, and the median age was 51 years (range, 0–94). Bacteremia without focus was the most common syndrome in 32 (38.6%) of the 83 cases, followed by bacteremic cellulitis (19.3%), bacteremic pneumonia (18.1%), and meningitis (7.2%).

Results for *emm* typing/serotyping were evaluable in 83 (100%) of the 83 cases by dWGS assay, and in 83 (100%) of the 83 cases by reference assay. Of *emm* type/capsule serotype results by reference assay, eight unique GAS *emm* types (including *emm* subtypes *emm*53.0 [*n* = 3], *emm*28.0 [*n* = 3], *emm*81.0 [*n* = 2], *emm*1.0 [*n* = 2], *emm*92.0, *emm*89.0, *emm*76.0, and *emm*151.1) were identified in 14 invasive GAS (iGAS) infections, six unique GBS serotypes (III [*n* = 10], IA [*n* = 8], II [*n* = 8], IB [*n* = 6], V [*n* = 5], and IV [*n* = 2]) in 39 invasive GBS (iGBS) infections, and 16 unique pneumococcal serotypes (3 [*n* = 4], 22F [*n* = 3], 23A [*n* = 3], 23B [*n* = 3], 8 [*n* = 3], 16F [*n* = 2], 19A [*n* = 2], 19F [*n* = 2], 15A, 22A, 35B/35D, 35F, 4, 6C, 7C, and 9N) in 30 invasive pneumococcal disease (IPD) cases. For *emm* type/capsule serotype, the percent agreement between dWGS assay results and reference assay was 100% (95% CI, 96% to 100%) (Table 1).

**TABLE 1:** Agreement between dWGS and reference assay on *emm*/capsular serotyping results

Pathogen	Cases^*a*^	Typing test	Unique types^*b*^	Percent agreement (95% CI)	PPA (95% CI)	NPA (95% CI)
GAS	14	*emm* typing	8	100% (77% to 100%)	100%	100%
GBS	39	Serotyping	6	100% (91% to 100%)	100%	100%
SPN	30	Serotyping	16	100% (88% to 100%)	100%	100%
All 3	83		30	100% (96% to 100%)	100%	100%

^*a*^ Cases with evaluable results from both dWGS and reference assay.

^*b*^ Based on reference assay results.

MLST results were evaluable in 81 (98%) of 83 cases in both dWGS and reference assay. The two non-evaluable results were in two GBS samples that had presumably novel alleles in the *glnA* and *adhP* locus, respectively. Among the 81 evaluable results in reference assay, 10 unique *S. pyogenes* MLSTs (11 [*n* = 3], 28 [*n* = 2], 52 [*n* = 2], 101, 433, 458, 50, 82, 837, and 909) were identified in 14 iGAS infections; 13 unique *S. agalactiae* MLSTs (23 [*n* = 8], 1 [*n* = 7], 22 [*n* = 5], 17 [*n* = 4], 12 [*n* = 2], 19 [*n* = 2], 459 [*n* = 2], 8 [*n* = 2], 10, 529, 596, 860, and 994) in 37 iGBS infections; and, 21 unique *S. pneumoniae* MLSTs (180 [*n* = 4], 10,148 [*n* = 2], 1,373 [*n* = 2], 338 [*n* = 2], 654 [*n* = 2], 698 [*n* = 2], 1,268, 1,451, 1,480, 1,635, 1,797, 18,248, 2,213, 2,829, 320, 36, 3811, 433, 53, 558, 6,029, and 639) in 30 IPD cases. For MLST, the percent agreement between dWGS and reference assay was 100% (95% CI, 96% to 100%) (Table 2). Type-specific PPA and NPA for *emm* typing, serotyping, and MLST are listed in Table S5.

**TABLE 2:** Agreement between dWGS and reference assay on MLST results

Pathogen	Cases^*a*^	Typing test	Unique types^*b*^	Percent agreement (95% CI)	PPA	NPA
GAS	14	MLST	10	100% (77% to 100%)	100%	100%
GBS	37	MLST	13	100% (91% to 100%)	100%	100%
SPN	30	MLST	22	100% (88% to 100%)	100%	100%
All 3	81		45	100% (96% to 100%)	100%	100%

^*a*^ Cases with evaluable results from both dWGS and reference assay.

^*b*^ Based on reference results.

For each pathogen identified in the 83 cases, antimicrobial susceptibility was predicted for 5 to 7 clinically relevant drugs depending on pathogen type. The total number of genotypic AST predictions performed was 489 (Table 3). Genotypic AST prediction results were evaluable in 487 (99.6%) of 489 tests in both dWGS and reference assay. The two non-evaluable results were in one GBS sample that had a presumably novel protein sequence type in PBP2× impacting genotypic AST prediction for penicillin and cefotaxime. Among evaluable results, 417 reference results (85.6%) were susceptible (S), 4 (0.8%) were intermediate (I), and 66 (13.6%) were resistant (R). The percent prediction agreement between dWGS and reference assay was 100% (487/487, 95% CI, 99% to 100%), FRP rate was 0% (0/417; 95% CI, 0% to 0.9%), and the FSP rate was 0% (0/66; 95% CI, 0% to 5.6%). The FIP rate was 0% (0/487; 95% CI, 0% to 0.8%).

**TABLE 3:** Agreement between dWGS and reference assay on genotypic AST prediction results

Pathogen	Cases^*a*^	Antimicrobial^*b*^	Evaluable results^*c*^	Prediction agreement^*d*^ (%; 95% CI)	FRP^*e*^ (%; 95% CI)	FSP^*f*^ (%; 95% CI)
GAS	14	CLI; ERY; PEN TAX; TET; VAN	84	84/84 (100%; 96%–100%)	0/80 (0%; 0%–4.5%)	0/4 (0%; 0%–60%)
GBS	39	CLI; ERY; PEN TAX; VAN	193	193/193 (100%; 98%–100%)	0/151 (0%; 0%–2.4%)	0/42 (0%; 0%–8.4%)
SPN	30	CLI; COT; ERY; LFX; PEN; TAX; TET; VAN	210	210/210 (100%; 98%–100%)	0/186 (0%; 0%–2.0%)	0/20 (0%; 5.3%–17%)
All 3	83		487	487/487 (100%; 99%–100%)	0/417 (0%; 0%–0.9%)	0/66 (0%; 0%–5.4%)

^*a*^ Case with evaluable genotypic AST prediction results from both dWGS and reference assay for one or more antimicrobials listed.

^*b*^ CLI: Clindamycin; COT: Trimethoprim-sulfamethoxazole; ERY: Erythromycin; LFX: Levofloxacin; PEN: Penicillin; TAX: Cefotaxime; TET: Tetracycline; VAN: Vancomycin.

^*c*^ Evaluable dWGS results accompanied by evaluable reference results for all antimicrobials listed.

^*d*^ The categorical result (S, I, or R) of dWGS assay is the same as that of the reference assay.

^*e*^ The reference result is S and the dWGS result is R.

^*f*^ The reference result is R and the dWGS result is S.

The draft genome assemblies that passed the dWGS QC criteria (*n* = 83) were compared with draft genome assemblies generated from matched isolates as reference to evaluate assembly quality (Table 4). There was no evidence that dWGS assemblies had lower quality metrics. Overall, the contig N50 of dWGS assemblies (mean ± SD, 123 ± 69 kb) was higher compared with that in reference assemblies (103 ± 56 kb; *P* = 0.02, Wilcoxon signed-rank test), indicating a higher continuity of dWGS genome assemblies. Consistent with this observation, dWGS assemblies had lower number of contigs (68 ± 56 vs 76 ± 45; *P* = 0.006, Wilcoxon signed-rank test) and a slightly higher assembly length (2.034 ± 0.118 Mb vs. 2.031 ± 0.117 Mb; *P* < 0.001, Wilcoxon signed-rank test). The SNP difference between a dWGS assembly and its matched reference assembly was 1.00 ± 0.00 (mean ± SD) SNPs per genome for GAS, 1.58 ± 1.42 for GBS, and 1.53 ± 0.97 for SPN.

**TABLE 4:** Comparison between dWGS and reference assay on draft genome assembly metrics

Assembly metrics	Pathogen	dWGS (mean ± SD)	Reference (mean ± SD)
Number of contigs	GAS (*n* = 14)	49 ± 23	71 ± 71
	GBS (*n* = 39)	52 ± 31	67 ± 40
	SPN (*n* = 30)	96 ± 79	90 ± 33
	All 3 (*n* = 83)	68 ± 56	76 ± 45
N50 (kb)	GAS (*n* = 14)	132 ± 66	114 ± 54
	GBS (*n* = 39)	139 ± 59	127 ± 62
	SPN (*n* = 30)	99 ± 78	67 ± 22
	All 3 (*n* = 83)	123 ± 69	103 ± 56
Assembly length (Mb)	GAS (*n* = 14)	1.81 ± 0.05	1.81 ± 0.06
	GBS (*n* = 39)	2.09 ± 0.06	2.08 ± 0.06
	SPN (*n* = 30)	2.07 ± 0.05	2.07 ± 0.05
	All 3 (*n* = 83)	2.03 ± 0.12	2.03 ± 0.12
SNP difference (mean ± SD)	GAS (*n* = 14)	0.29 ± 0.47	
	GBS (*n* = 39)	1.23 ± 2.12	
	SPN (*n* = 30)	1.27 ± 1.20	
	All 3 (*n* = 83)	1.08 ± 1.68	

We investigated the 14 blood culture samples that failed to pass the dWGS QC criteria (Table S2). The pathogen-specific cycle threshold (Ct) value of the 14 failed samples (mean ± SD: 23.1 ± 6.4) was significantly higher than that of the 83 samples that passed dWGS QC (16.5 ± 2.1; *P* = 0.002, *t*-test). In addition, the sample storage time (days between positive culture and DNA extraction) of the 14 failed samples (164 ± 60 days) was significantly longer than that of the 83 samples that passed dWGS QC (113 ± 51; *P* = 0.009, *t*-test). The results suggested that lower levels of microbial DNA in some blood culture samples, partially due to degradation during longer storage time, may have contributed to the 14% dWGS genome assembly failure rate. It should be noted that the QC failures were post-sequencing assembly failures, which were used as a primary end point for WGS QC. In addition to pathogen-specific Ct values, we investigated two other pre-sequencing QC criteria, the DNA and library concentrations. The DNA concentrations did not differ significantly between the 14 failed samples (11.1 ± 14.4 ng/µL) and the 83 samples that passed dWGS QC (10.4 ± 7.2 ng/µL, *P* = 0.87, *t*-test), nor did the library concentrations (2.3 ± 3.8 nM vs 3.9 ± 2.6 nM, *P* = 0.17, *t*-test), suggesting that DNA and library concentrations were less informative than pathogen-specific Ct as QC predictors. When the 14 samples were included in comparison, the percent agreement was 95% (92/97; 95% CI, 88% to 98%) for *emm*/serotyping, 90% (87/97; 95% CI, 82% to 94%) for MLST, and 95% (546/576; 95% CI, 93% to 96%) for AST categorical results.

## DISCUSSION

In this study, we demonstrated the performance of the dWGS assay, which allows WGS-based pathogen characterization directly from blood culture samples without isolation of pure cultured isolates. For 83 (86%) of 97 blood culture samples positive for GAS, GBS, or SPN, the dWGS assay generated whole-genome assemblies that passed the assay QC criteria and demonstrated equal or better assembly metrics and minimum SNP difference (error rate of approximately 1 × 10^−6^) compared with what were obtained from the reference, isolate-based WGS procedures. The dWGS assay also produced pathogen features, including *emm* types, capsular serotypes, MLST, and genotypic AST prediction, that were 100% identical to the reference results for all samples that passed QC. These results indicate that the dWGS assay has a great potential to be used by public health laboratories for disease surveillance and outbreak response, benefiting from its ability to directly utilize blood culture samples that are often tested for pathogen identification in clinical laboratories. Additionally, the dWGS assay could cut turn-around time and reduce WGS costs through eliminating the time and laboratory materials required for sub-culture and strain isolation.

For 14 (14%) of 97 positive blood culture samples, the dWGS assay failed to generate whole-genome assemblies passing the assay QC criteria. GAS blood culture samples exhibited a much lower rate of WGS QC failure (1/15, 6.7%) compared with SPN samples (8/38, 21%). These 14 samples, although all confirmed to be positive for GAS, GBS, or SPN as reported by clinical laboratories, generally exhibited lower levels of bacterial DNA (higher pathogen-specific Ct values) compared with samples that passed the assay QC. The results suggested the dWGS assay could benefit from improving microbial DNA recovery from samples that also contain human DNA prior to the WGS procedures. Potential methods for improved microbial DNA recovery include differential cell lysis (31), multiple displacement amplification (MDA) of bacterial whole genome (32), and RNA bait-based target DNA enrichment (33). Because these additional sample processing steps could significantly increase the cost and turnaround time for the dWGS assay, they need to be evaluated against the potential benefit of a higher assay success rate, which varies by pathogen.

This study had several limitations. First, evaluation of the dWGS assay was limited to a relatively small number of samples (*n* = 97) from a single U.S. state. A larger evaluation with more diverse samples is needed to validate the assay performance. Second, the performance of the dWGS for pathogens other than GAS, GBS, and SPN needs to be further explored. Another limitation was that mixed infections or contaminating organisms were not assessed by using a metagenome-aware assembler or read classification. Nonetheless, for these streptococcal pathogens, the dWGS assay delivered essentially equivalent results as the isolate-based WGS without the time and expense required for sub-culture. With further validation and improvement, the dWGS assay could better serve public health laboratories in the metagenomics era. It should be noted that clinical isolates remain uniquely valuable for confirmatory phenotypic assays, archiving, and other public health or clinical functions. Currently, the risk of not recovering clinical isolates from blood cultures is low. With further refinement, the WGS methods used in this study could also help address decreased isolates submission due to increasing use of culture-independent diagnostic tests (10).

### Summary

For 86% of blood culture samples, a dWGS assay delivered nearly identical results of streptococcal strain characterization and whole-genome assembly as isolate-based WGS, without the time and expense required for sub-culture and isolation.

## Data Availability

WGS data have been deposited at the National Center for Biotechnology Information Sequence Read Archive with accession numbers PRJNA395240 (GAS), PRJNA355303 (GBS), and PRJNA284954 (SPN).

## SUPPLEMENTAL MATERIAL

Supplemental Material jcm.01126-25-s0001.pdf Tables S1 to S5.

ASM does not own the copyrights to Supplemental Material that may be linked to, or accessed through, an article. The authors have granted ASM a non-exclusive, world-wide license to publish the Supplemental Material files. Please contact the corresponding author directly for reuse.
