# Supplementary material for: Whole-genome sequencing-based pathogen characterization for streptococcal infection directly from positive blood culture samples
Source: J Clin Microbiol. 2025 Dec 8;64(1):e01126-25. doi: 10.1128/jcm.01126-25 (PMC12802199; doi:10.1128/jcm.01126-25)
Supplement: Supplemental Material — Tables S1 to S5. [file jcm.01126-25-s0001.pdf]

Table S1. List of tests performed

**Pathogen**	**Test**
GAS	*emm* typing<br>Multilocus Sequence Typing<br>Genotypic AST prediction[1]:<br>CLI<br>ERY<br>PEN<br>TAX<br>TET<br>VAN
GBS	Serotyping<br>Multilocus Sequence Typing<br>Genotypic AST prediction[1]:<br>CLI<br>ERY<br>PEN<br>TAX<br>VAN
SPN	Serotyping<br>Multilocus Sequence Typing<br>Genotypic AST prediction[1]:<br>CLI<br>COT<br>ERY<br>LFX<br>PEN<br>TAX<br>TET<br>VAN

1. CLI: Clindamycin; COT: Trimethoprim-sulfamethoxazole; ERY: Erythromycin; LFX: Levofloxacin; PEN: Penicillin; TAX: Cefotaxime; TET: Tetracycline; VAN: Vancomycin

Table S2. Pathogen-specific Ct values and sample storage time for all 97 eligible blood culture samples in this study

LABID	Pathogen	Ct	Storage Time (Days)	dWGS Assembly Passed QC
035885-22	GBS	16.32	173	NO
035881-22	GBS	16.36	208	NO
035887-22	GBS	17.54	159	NO
5569-21	GAS	17.75	32	NO
035868-22	SPN	19.66	178	NO
035884-22	GBS	20.49	183	NO
035861-22	SPN	22.01	243	NO
035865-22	SPN	22.22	209	NO
035860-22	SPN	22.28	245	NO
035866-22	SPN	24.5	196	NO
041372-22	SPN	25.7	113	NO
035875-22	SPN	26.27	138	NO
0123-22	GBS	35.67	83	NO
035876-22	SPN	36.71	130	NO
0124-22	GBS	6.39	83	YES
041375-22	GAS	12.13	88	YES
0104-22	SPN	13.495	118	YES
0103-22	SPN	13.785	122	YES
5564-21	SPN	14.01	58	YES
0113-22	GAS	14.51	47	YES
0100-22	SPN	14.53	140	YES
041376-22	GAS	14.55	70	YES
0099-22	SPN	14.825	148	YES
5580-21	GBS	14.92	42	YES
0108-22	SPN	15.12	68	YES
0128-22	GBS	15.155	42	YES
5572-21	GBS	15.16	73	YES
0109-22	GAS	15.26	104	YES
5578-21	GBS	15.37	52	YES
5563-21	SPN	15.37	59	YES
0112-22	GAS	15.43	66	YES
035888-22	GBS	15.46	152	YES
0120-22	GBS	15.5	105	YES
0106-22	SPN	15.5	101	YES
0119-22	GBS	15.625	107	YES
0111-22	GAS	15.63	92	YES
0115-22	GBS	15.645	135	YES

0125-22	GBS	15.685	72	YES
5573-21	GBS	15.73	73	YES
035886-22	GBS	15.79	172	YES
0116-22	GBS	15.795	129	YES
0117-22	GBS	15.895	121	YES
0110-22	GAS	15.93	87	YES
5579-21	GBS	16	44	YES
0114-22	GBS	16.005	148	YES
041368-22	SPN	16.02	146	YES
0118-22	GBS	16.035	112	YES
5574-21	GBS	16.04	71	YES
0105-22	SPN	16.07	119	YES
5576-21	GBS	16.13	57	YES
035883-22	GBS	16.14	188	YES
0122-22	GBS	16.16	87	YES
0127-22	GBS	16.165	40	YES
041377-22	GBS	16.18	183	YES
035882-22	GBS	16.21	205	YES
0121-22	GBS	16.295	99	YES
5577-21	GBS	16.4	55	YES
5565-21	SPN	16.4	60	YES
035889-22	GBS	16.41	120	YES
5575-21	GBS	16.43	71	YES
041380-22	GBS	16.47	90	YES
041383-22	GBS	16.52	43	YES
035880-22	GBS	16.54	211	YES
041382-22	GBS	16.62	82	YES
035891-22	GBS	16.64	106	YES
0126-22	GBS	16.655	72	YES
041381-22	GBS	16.66	103	YES
5566-21	SPN	16.73	24	YES
035877-22	GAS	16.74	195	YES
041378-22	GBS	16.77	176	YES
035890-22	GBS	16.78	110	YES
041379-22	GBS	16.81	98	YES
035863-22	SPN	16.88	217	YES
5568-21	GAS	16.97	69	YES
035892-22	GBS	17.09	105	YES
035872-22	SPN	17.27	162	YES
041371-22	SPN	17.27	128	YES
041367-22	SPN	17.33	148	YES

5567-21	GAS	17.7	76	YES
035873-22	SPN	17.81	149	YES
5571-21	GAS	17.92	28	YES
041369-22	SPN	17.92	130	YES
035862-22	SPN	17.98	240	YES
041364-22	SPN	18.04	179	YES
5570-21	GAS	18.7	29	YES
035869-22	SPN	18.71	177	YES
041362-22	SPN	18.71	213	YES
041365-22	SPN	18.99	166	YES
035879-22	GAS	19.01	120	YES
035874-22	SPN	19.26	139	YES
035867-22	SPN	19.37	178	YES
035871-22	SPN	19.95	171	YES
0102-22	SPN	20.54	130	YES
035878-22	GAS	20.57	171	YES
041366-22	SPN	21.03	149	YES
041373-22	SPN	21.55	83	YES
035870-22	SPN	21.94	172	YES

Table S3. The number of human reads removed from each blood culture sample.

LABID	Number of Reads Before Human Reads removal	Number of Reads Removed	Proportion removed
0099-22	1049890	711625	67.8%
0100-22	812756	518961	63.9%
0102-22	2253457	1622334	72.0%
0103-22	1140510	518014	45.4%
0104-22	747246	87482	11.7%
0105-22	906622	290895	32.1%
0106-22	1235645	54448	4.4%
0108-22	874124	197190	22.6%
0109-22	1084155	255160	23.5%
0110-22	595870	68737	11.5%
0111-22	1026845	217321	21.2%
0112-22	833754	971	0.1%
0113-22	939937	19762	2.1%
0114-22	1195276	279606	23.4%
0115-22	869260	263522	30.3%
0116-22	1272349	901435	70.8%
0117-22	895572	347128	38.8%
0118-22	1053082	47411	4.5%
0119-22	994073	445052	44.8%
0120-22	811679	399651	49.2%
0121-22	1159382	870	0.1%
0122-22	1363524	423901	31.1%
0123-22	2314900	1909070	82.5%
0124-22	657312	248881	37.9%
0125-22	2507451	2113484	84.3%
0126-22	2913689	2437605	83.7%
0127-22	886048	167355	18.9%
0128-22	757902	485042	64.0%
035860-22	1623209	500371	30.8%
035861-22	2140740	2065137	96.5%
035862-22	1930157	177242	9.2%
035863-22	1279413	754223	59.0%
035865-22	2471974	230065	9.3%
035866-22	345762	117882	34.1%
035867-22	1941479	808745	41.7%
035868-22	1921444	1277916	66.5%
035869-22	2481129	1336141	53.9%

035870-22	1869596	1233388	66.0%
035871-22	1157954	307181	26.5%
035872-22	2638966	1135077	43.0%
035873-22	1014062	440159	43.4%
035874-22	1996576	491610	24.6%
035875-22	190583	131384	68.9%
035876-22	1194538	951436	79.6%
035877-22	1207868	22391	1.9%
035878-22	1972484	1752765	88.9%
035879-22	1594543	4539	0.3%
035880-22	1567784	110530	7.1%
035881-22	1916166	1808545	94.4%
035882-22	2144569	1415992	66.0%
035883-22	1550605	985897	63.6%
035884-22	2229950	1299598	58.3%
035885-22	1817293	1321087	72.7%
035886-22	1527062	630006	41.3%
035887-22	1709910	1448556	84.7%
035888-22	1590064	982902	61.8%
035889-22	1823433	170918	9.4%
035890-22	1401710	787467	56.2%
035891-22	1792240	1043561	58.2%
035892-22	1953939	567436	29.0%
041362-22	1180269	675034	57.2%
041364-22	1020279	342235	33.5%
041365-22	1010881	231575	22.9%
041366-22	1125804	534868	47.5%
041367-22	669233	140125	20.9%
041368-22	1025687	230129	22.4%
041369-22	1120782	359225	32.1%
041371-22	1099070	267489	24.3%
041372-22	335126	82315	24.6%
041373-22	1479313	877865	59.3%
041375-22	1324149	253248	19.1%
041376-22	930963	1860	0.2%
041377-22	771510	1239	0.2%
041378-22	791697	425214	53.7%
041379-22	1427692	1053857	73.8%
041380-22	956850	566774	59.2%
041381-22	832637	35039	4.2%
041382-22	1688003	1304467	77.3%

041383-22	1965349	660744	33.6%
5563-21	1569714	704372	44.9%
5564-21	1228451	895125	72.9%
5565-21	1615549	972679	60.2%
5566-21	1030726	758947	73.6%
5567-21	4709823	12890	0.3%
5568-21	722955	139450	19.3%
5569-21	2328952	12625	0.5%
5570-21	1292460	4463	0.3%
5571-21	1556967	4924	0.3%
5572-21	2924550	2558759	87.5%
5573-21	1166705	863279	74.0%
5574-21	907497	269924	29.7%
5575-21	2916475	737915	25.3%
5576-21	1715771	428565	25.0%
5577-21	594049	80410	13.5%
5578-21	805183	488677	60.7%
5579-21	759184	45320	6.0%
5580-21	1230042	969128	78.8%

Table S4. Demographic characteristics and syndromes of ABCs cases whose blood culture samples were included in the dWGS based pathogen characterization.

	All	GAS	GBS	SPN
**Number of Cases**	83	14	39	30
**Age**				
**<5 years**	28(33.7%)	1(7.1%)	13(33.3%)	14(46.7%)
**5-17 years**	5(6.0%)	1(7.1%)	0(0.0%)	4(13.3%)
**18-64 years**	19(22.9%)	6(42.9%)	8(20.5%)	5(16.7%)
**> 64 years**	31(37.3%)	6(42.9%)	18(46.2%)	7(23.3%)
**Sex**				
**Female**	31(37.3%)	5(35.7%)	15(38.5%)	11(36.7%)
**Male**	52(62.7%)	9(64.3%)	24(61.5%)	19(63.3%)
**Syndromes[1]**				
**BACTSYN**	32(38.6%)	2(14.3%)	20(51.3%)	10(33.3%)
**CELLSYN**	15(18.1%)	6(42.9%)	9(23.1%)	0(0.0%)
**PNEUSYN**	15(18.1%)	0(0.0%)	0(0.0%)	15(50.0%)
**MENSYN**	6(7.2%)	0(0.0%)	2(5.1%)	4(13.3%)
**OSTEOSYN**	3(3.6%)	2(14.3%)	1(2.6%)	0(0.0%)
**OTHSYN**	3(3.6%)	0(0.0%)	2(5.1%)	1(3.3%)
**SESHKSYNONLY**	3(3.6%)	0(0.0%)	3(7.7%)	0(0.0%)
**ARTHSYN**	2(2.4%)	2(14.3%)	0(0.0%)	0(0.0%)
**SESHKSYN**	2(2.4%)	0(0.0%)	2(5.1%)	0(0.0%)
**OTITSYN**	1(1.2%)	1(7.1%)	0(0.0%)	0(0.0%)
**STSSSYN**	1(1.2%)	1(7.1%)	0(0.0%)	0(0.0%)

1. BACTSYN: Bacteremia without focus; CELLSYN: Cellulitis; PNEUSYN: Pneumonia; MENSYN: Meningitis; OSTEOSYN: Osteomyelitis; OTHSYN: Other syndrome; SESHKSYNONLY: Only septic shock is checked for syndrome; ARTHSYN: Septic arthritis; SESHKSYN: Septic Shock; OTITSYN: Otitis media; STSS: Streptococcal Toxic Shock Syndrome.

Table S5. Type-specific positive percent agreement (PPA) and negative percent agreement (NPA) for *emm* typing, serotyping, and MLST

PATHOGEN	TEST	N_CASE	REF_TYPE	N_TYPE	TP	TN	FP	FN	PPA	PPA_LCI	PPA_UCI	NPA	NPA_LCI	NPA_UCI
GAS	GAS_*emm*	14	28.0	3	3	11	0	0	1	0.29	1	1	0.72	1
GAS	GAS_*emm*	14	89.0	1	1	13	0	0	1	0.03	1	1	0.75	1
GAS	GAS_*emm*	14	53.0	3	3	11	0	0	1	0.29	1	1	0.72	1
GAS	GAS_*emm*	14	81.0	2	2	12	0	0	1	0.16	1	1	0.74	1
GAS	GAS_*emm*	14	76.0	1	1	13	0	0	1	0.03	1	1	0.75	1
GAS	GAS_*emm*	14	151.1	1	1	13	0	0	1	0.03	1	1	0.75	1
GAS	GAS_*emm*	14	1.0	2	2	12	0	0	1	0.16	1	1	0.74	1
GAS	GAS_*emm*	14	92.0	1	1	13	0	0	1	0.03	1	1	0.75	1
GAS	GAS_MLST	14	458	1	1	13	0	0	1	0.03	1	1	0.75	1
GAS	GAS_MLST	14	101	1	1	13	0	0	1	0.03	1	1	0.75	1
GAS	GAS_MLST	14	11	3	3	11	0	0	1	0.29	1	1	0.72	1
GAS	GAS_MLST	14	52	2	2	12	0	0	1	0.16	1	1	0.74	1
GAS	GAS_MLST	14	837	1	1	13	0	0	1	0.03	1	1	0.75	1
GAS	GAS_MLST	14	909	1	1	13	0	0	1	0.03	1	1	0.75	1
GAS	GAS_MLST	14	50	1	1	13	0	0	1	0.03	1	1	0.75	1
GAS	GAS_MLST	14	433	1	1	13	0	0	1	0.03	1	1	0.75	1
GAS	GAS_MLST	14	28	2	2	12	0	0	1	0.16	1	1	0.74	1
GAS	GAS_MLST	14	82	1	1	13	0	0	1	0.03	1	1	0.75	1
GBS	GBS_MLST	37	1	7	7	30	0	0	1	0.59	1	1	0.88	1
GBS	GBS_MLST	37	23	8	8	29	0	0	1	0.63	1	1	0.88	1
GBS	GBS_MLST	37	17	4	4	33	0	0	1	0.40	1	1	0.89	1
GBS	GBS_MLST	37	8	2	2	35	0	0	1	0.16	1	1	0.90	1
GBS	GBS_MLST	37	22	5	5	32	0	0	1	0.48	1	1	0.89	1
GBS	GBS_MLST	37	12	2	2	35	0	0	1	0.16	1	1	0.90	1
GBS	GBS_MLST	37	19	2	2	35	0	0	1	0.16	1	1	0.90	1
GBS	GBS_MLST	37	459	2	2	35	0	0	1	0.16	1	1	0.90	1
GBS	GBS_MLST	37	596	1	1	36	0	0	1	0.03	1	1	0.90	1
GBS	GBS_MLST	37	860	1	1	36	0	0	1	0.03	1	1	0.90	1
GBS	GBS_MLST	37	10	1	1	36	0	0	1	0.03	1	1	0.90	1
GBS	GBS_MLST	37	529	1	1	36	0	0	1	0.03	1	1	0.90	1
GBS	GBS_MLST	37	994	1	1	36	0	0	1	0.03	1	1	0.90	1
GBS	GBS_SEROTYPE	39	V	5	5	34	0	0	1	0.48	1	1	0.90	1
GBS	GBS_SEROTYPE	39	IA	8	8	31	0	0	1	0.63	1	1	0.89	1
GBS	GBS_SEROTYPE	39	III	10	10	29	0	0	1	0.69	1	1	0.88	1
GBS	GBS_SEROTYPE	39	IB	6	6	33	0	0	1	0.54	1	1	0.89	1
GBS	GBS_SEROTYPE	39	II	8	8	31	0	0	1	0.63	1	1	0.89	1
GBS	GBS_SEROTYPE	39	IV	2	2	37	0	0	1	0.16	1	1	0.91	1
SPN	SPN_MLST	30	10148	2	2	28	0	0	1	0.16	1	1	0.88	1

SPN	SPN_MLST	30	698	2	2	28	0	0	1	0.16	1	1	0.88	1
SPN	SPN_MLST	30	18248	1	1	29	0	0	1	0.03	1	1	0.88	1
SPN	SPN_MLST	30	2213	1	1	29	0	0	1	0.03	1	1	0.88	1
SPN	SPN_MLST	30	639	1	1	29	0	0	1	0.03	1	1	0.88	1
SPN	SPN_MLST	30	2829	1	1	29	0	0	1	0.03	1	1	0.88	1
SPN	SPN_MLST	30	1797	1	1	29	0	0	1	0.03	1	1	0.88	1
SPN	SPN_MLST	30	180	4	4	26	0	0	1	0.40	1	1	0.87	1
SPN	SPN_MLST	30	3811	1	1	29	0	0	1	0.03	1	1	0.88	1
SPN	SPN_MLST	30	1635	1	1	29	0	0	1	0.03	1	1	0.88	1
SPN	SPN_MLST	30	654	2	2	28	0	0	1	0.16	1	1	0.88	1
SPN	SPN_MLST	30	338	2	2	28	0	0	1	0.16	1	1	0.88	1
SPN	SPN_MLST	30	36	1	1	29	0	0	1	0.03	1	1	0.88	1
SPN	SPN_MLST	30	320	1	1	29	0	0	1	0.03	1	1	0.88	1
SPN	SPN_MLST	30	1451	1	1	29	0	0	1	0.03	1	1	0.88	1
SPN	SPN_MLST	30	1373	2	2	28	0	0	1	0.16	1	1	0.88	1
SPN	SPN_MLST	30	1268	1	1	29	0	0	1	0.03	1	1	0.88	1
SPN	SPN_MLST	30	558	1	1	29	0	0	1	0.03	1	1	0.88	1
SPN	SPN_MLST	30	53	1	1	29	0	0	1	0.03	1	1	0.88	1
SPN	SPN_MLST	30	6029	1	1	29	0	0	1	0.03	1	1	0.88	1
SPN	SPN_MLST	30	1480	1	1	29	0	0	1	0.03	1	1	0.88	1
SPN	SPN_MLST	30	433	1	1	29	0	0	1	0.03	1	1	0.88	1
SPN	SPN_SEROTYPE	30	16F	2	2	28	0	0	1	0.16	1	1	0.88	1
SPN	SPN_SEROTYPE	30	22F	3	3	27	0	0	1	0.29	1	1	0.87	1
SPN	SPN_SEROTYPE	30	9N	1	1	29	0	0	1	0.03	1	1	0.88	1
SPN	SPN_SEROTYPE	30	4	1	1	29	0	0	1	0.03	1	1	0.88	1
SPN	SPN_SEROTYPE	30	6C	1	1	29	0	0	1	0.03	1	1	0.88	1
SPN	SPN_SEROTYPE	30	23A	3	3	27	0	0	1	0.29	1	1	0.87	1
SPN	SPN_SEROTYPE	30	7C	1	1	29	0	0	1	0.03	1	1	0.88	1
SPN	SPN_SEROTYPE	30	3	4	4	26	0	0	1	0.40	1	1	0.87	1
SPN	SPN_SEROTYPE	30	15A	1	1	29	0	0	1	0.03	1	1	0.88	1
SPN	SPN_SEROTYPE	30	35F	1	1	29	0	0	1	0.03	1	1	0.88	1
SPN	SPN_SEROTYPE	30	19F	2	2	28	0	0	1	0.16	1	1	0.88	1
SPN	SPN_SEROTYPE	30	23B	3	3	27	0	0	1	0.29	1	1	0.87	1
SPN	SPN_SEROTYPE	30	19A	2	2	28	0	0	1	0.16	1	1	0.88	1
SPN	SPN_SEROTYPE	30	8	3	3	27	0	0	1	0.29	1	1	0.87	1
SPN	SPN_SEROTYPE	30	35B:35D	1	1	29	0	0	1	0.03	1	1	0.88	1
SPN	SPN_SEROTYPE	30	22A	1	1	29	0	0	1	0.03	1	1	0.88	1
